# The relationship between social support and academic engagement among university students: the chain mediating effects of life satisfaction and academic motivation

**DOI:** 10.1186/s12889-023-17301-3

**Published:** 2023-11-29

**Authors:** Chunmei Chen, Fei Bian, Yujie Zhu

**Affiliations:** 1 Teachers College, Jimei University, Xiamen, Fujian 361021 China; 2 Institute of Technical and Vocational Education, Shenzhen Polytechnic University, Shenzhen, Guangdong 518055 China; 3 School of Marine Culture and Law, Jimei University, Xiamen, Fujian 361021 China

**Keywords:** University students, Social support, Academic engagement, Life satisfaction, Academic motivation

## Abstract

**Background:**

University students’ academic engagement has a significant impact on their academic performance and career development.

**Methods:**

In order to explore the influential mechanisms of social support on university students’ academic engagement and the mediating role of academic motivation and life satisfaction, this study used the Adolescent Social Support Scale, University Students’ Academic Engagement Scale Questionnaire, Adolescent Student Life Satisfaction Scale and University Students’ Academic Motivation Questionnaire, to conduct a questionnaire survey and empirical analysis on 2106 Chinese university students.

**Results:**

(1) social support significantly and positively predicts academic engagement; (2) social support influences academic engagement through the mediating effect of life satisfaction; (3) social support influences academic engagement through the mediating effect of academic motivation; (4) life satisfaction and academic motivation play a chain mediating role in the effect of social support on academic engagement.

**Conclusions:**

This study contributes to understanding the underlying mechanisms of the relationship between social support and academic engagement, which in turn provides insights for universities and the departments concerned to make measures to improve the level of university students’ academic engagement.

## Introduction

According to the Ministry of Education of the People’s Republic of China, as of 2022, there was a total of 3,013 higher education institutions in the country. Among them, there were 1,239 general undergraduate schools with a total of 19,656,400 students enrolled [1]. As a main force for buiding the country, university students however more and more commonly lack academic engagement in learning. This might pose a risk to the cultivation of undergraduate talents in China [2]. Considering that the level of students’ academic engagement in higher education institutions is increasingly recognized as a valid indicator of institutional excellence [3]. Thus, one key factor to higher education development is to improve university students’ academic engagement, which in turn enhances the quality of talent cultivation in undergraduate universities [4]. Academic engagement reflects the quality of students’ participation, investment, commitment into and recognition with schools and related activities to improve students’ performance [5]. It is the extent to which students are committed to schools and are motivated to learn [6]. Only when students are actively engaged in the learning process can they have meaningful and lasting learning experiences [7, 8]. Academic engagement encompasses the behavioral (e.g., participation in academic and social activities), affective (comprised of students’ attitudes, interests, and values), and cognitive (e.g., motivational goals and the application of learning strategies, etc.) dimensions of an individual’s engagement in the learning process [9]. More specifically, academic engagement includes students attending classes, completing assignments, interacting with peers and instructors, and enrolling and participating in extracurricular activities [10]. Academic engagement also refers to the time and efforts that students invest into the activities with educational purposes [11]. Academic engamenment is typically characterized as vitality (representing energy, willingness, and persistence in the face of difficulties), dedication (understanding the meaning of the work, being enthusiastic, inspired, and proud of the work), and absorption (focusing on the work) [12]. High levels of academic engagement are necessary for students’ success in universities [13, 14]. Scholars have confirmed that university students’ learning effectiveness depends on their academic engagement [15]. Their academic engagement is a better predictor to students’ learning outcomes such as critical thinking, cognitive activities, and reading and writing skills [16]. Research into university students’ academic engagement can help reduce dissatisfaction, avoid boredom, increase motivation to participate in school-related activities, and improve their achievement level [17, 18]. University students’ academic engagement has become a topic of wide interest and discussion in the academic community. However, Studies in Fujian and Zhejiang provinces of of China have found that the overall level of university students’ academic engagement is on an average or even moderately low level [19, 20]. It has been found that situational awareness, academic motivation, affective input, cognitive input, behavioral input and learning gain constitute a learning input mechanism that influences and constrains each other [21]. Based on 134,178 undergraduate students from 311 universities in China, a study survey has used the self-system model of motivational development as a theoretical framework.to examine the internal and external influencing mechanisms of university students’ academic engagement [22]. Another investigation of the factors influencing the lack of academic engagement of some university students in four higher education institutions in Jiangxi Province has found that there is a significant positive correlation between active cooperation, learning attitude, family factors, teaching management, school support and university students’ academic engagement [23]. In addition, scholars have explored the impact of instructor support strategies on university students’ academic engagement in online learning. University students’ academic engagement may be related to social support, life satisfaction, and academic motivation [24]. What factors are associated with university students’ academic engagement? How do these factors affect academic engagement? This study aims to investigate the relationship between university students’ academic engagement and these factors and the mechanism of their influence.

### Relationship between social support and learning engagement

Social support refers to the social and psychological support that an individual receives or perceives in the environment, such as respect, care and help [25]. Psychological support refers mainly to the emotional and evaluative support provided [26], whereas non-psychological support refers mainly to instrumental and material support [27]. Evaluation support is the most common and frequently occurring social support among university students. Social support for classroom evaluation is positively correlated with academic engagement [28]. In addition, technology-supported learning environments promote greater classroom participation [29]. Students’ need for relevance or belonging is viewed as the extent to which students feel accepted and supported by teachers and peers [30]. This is even more important at the university level because students are often faced with the need to establish and maintain new relationships as their transition from high school to universities [31]. According to Hernandez et al. (2021), social support refers to any assistance and help provided to someone by others. Positive relationships are built around the provision of these supports. In educational settings, the groups that give social support are usually teachers, peers and parents. Through their research, they find that social support has a positive impact on university students’ academic engagement [32]. Guardian support helps students to be engaged in learning and achieve better academic success [33]. Moreover, good relationships with peers and teachers increase the likelihood that adolescents would demonstrate higher level of behavioral engagement in the classroom [34]. In particular, the social support provided by teachers that students find accessible plays a key role in the maintenance and development of students’ academic engagement [35]. Social support is positively related to academic engagement [36–38]. In summary, hypothesis H1 is proposed.

#### H1

There is a significant effect of social support on learning engagement.

### Mediating effects of life satisfaction

Life satisfaction is an important cognitive measure of subjective well-being, which refers to an individual’s overall cognitive assessment of his or her life situation most of the time or over a certain period of time according to the criteria chosen by himself or herself. It is an important parameter of an individual’s life quality in a given society since it offers an overall perception and evaluation of life quality on a positive-to-negative continuum, and is an important parameter of the quality of life of individuals in a given society [39]. According to Restubog et al. (2010), social support is a form of social capital that is acquired through social interactions in various human groups. General social support from family, friends, and significant others can contribute to the development of an individual’s life satisfaction [40]. The relationship between different sources of social support (family, peers, and teachers) and life satisfaction among 1,133 Korean adolescents is investigated. It is found that the level of social support is one of the core factors of life satisfaction, which is positively correlated with life satisfaction [41]. It has been established that social support is a buffer against psychological distress [42], a factor influencing mental health [43, 44], and a protective mechanism for life satisfaction [45], which is positively correlated with life satisfaction. In addition, life satisfaction is an important factor in mental and psychological well-being and is related to the individual’s perception of cheerfulness, which is significantly and positively correlated with students’ academic engagement [46]. This is in line with previous studies [47, 48]. Students with higher life satisfaction across dimensions (environment, family, school, peers, self) are more academically engaged [49]. Students’ life satisfaction affects their academic engagement. In summary, hypothesis H2 is proposed:

#### H2

Life satisfaction mediates the relationship between social support and academic engagement.

### The mediating effect of academic motivation

Academic motivation is the internal motivation that directly pushes students to learn, and it has an initiating, maintaining and orienting effect on learning. The nature and intensity of academic motivation directly affects the direction, progress and outcomes of university students’ learning [39]. Hsieh (2014) has pointed out that motivation consists of two dimensions: internal and external motivation. Internal motivations mainly refers to the students’ engagement in learning driven by challenges, curiosity and knowledge acquisition. External motivations represents external incentives such as grades, rewards and competitions with or evaluations from others [50]. There is a positive correlation between students’ academic motivation and the support they receive from their parents, teachers, and friends [51]. The sense of social support received from teacher-student and family relationships compensates for students’ daily low moods and thus positively affects their internal motivation to learn [52]. Camacho et al. (2021) have put forward that teachers’ social support perceived by students is a significant predictor of students’ motivation. The higher the teacher’s social support is, the lower students’ academic motivation declines [53]. It has been shown that students with higher level of social support have higher level of academic motivation [54]. In addition, students’ motivation has an impact on their academic engagement. Motivation is what drives students’ behavior, and engagement is explained through specific manifestations of students’ behavior or motivation [55]. Specific motivational structures can uniquely predict engagement [56, 57]. Numerous studies have confirmed that academic motivation is positively correlated with academic engagement [36, 58–60]. In summary, hypothesis H3 is proposed.

#### H3

Academic motivation mediates the relationship between social support and academic engagement.

### Chain-mediated effects of life satisfaction and academic motivation

Sense of social support can help adolescents connect their goals with those of others so that they begin to gain a shared understanding of how the world works. Students with a higher sense of social support show a higher level of life satisfaction [61]. Many students’ life satisfaction reduces their life satisfaction because they are disappointed in social relationships and the constructed social support system is not strong [62]. High life satisfaction allows flexibility and autonomy for young people to pursue higher education, as well as providing them the possibility of attending various events and opportunities for personal development. Life satisfaction is a positive predictor of increased academic motivation [63]. Previous study arrives at a similar view. Students with high life satisfaction tend to believe that relying on their own abilities and behaviors can largely reduce academic stress and activate academic motivation. They always have a full enthusiasm and passion for learning [64]. Numerous studies have confirmed that life satisfaction is positively correlated with academic motivation [65–67]. The higher the university students’ life satisfaction is, the higher their level of academic motivation becomes. Students with high level of academic motivation do not give up easily when they encounter difficulties in their studies, instead they usually sollutions, which enables them to acquire the behavioral and cognitive strategies, information, and emotional energy demanded for task re-engagement. Therefore,. these students have high academic engagement [68]. In summary, hypothesis H4 is proposed:

#### H4

Life satisfaction and academic motivation chain mediate the effect of social support on academic engagement among university students.

This study constructed a chain mediating model to examine the effects of social support on academic engagement and the mediating role of life satisfaction and academic motivation between the two in the group of university students, with a view to providing guidance for improving university students’ academic engagement.

## Research methodology

### Research subjects

Convenient sampling method was used to select subjects from several universities in China (Jimei University, Xiamen Institute of Technology, Xiamen Medical College, Guangdong University of Petrochemical Technology, etc.) to conduct a questionnaire survey, and 2,106 valid questionnaires were collected and sorted out. The age of the subjects ranged from 17 to 23 years old (M = 20.16 years old, SD = 1.31). The basic characteristics of the sample are shown in Table 1.

**Table 1:** Descriptive statistics of the sample

Name	Options	Frequency	Percentage(%)
Gender	male	1224	58.12
	female	882	41.88
Cultivation Level	freshman	1061	50.38
	sophomore	572	27.16
	junior	305	14.48
	senior	168	7.98
Total		2106	100.0

### Research instruments

#### Adolescent social support scale

The Adolescent Social Support Scale was developed by Ye et al. in 2008 [39]. The scale includes the social support resources that the respondent receives and his/her utilization of the available resources. The Adolescent Social Support Scale is a self-report scale including three dimensions: subjective support, objective support, and support utilization, with a total of 17 entries on a five-point scale. All item scores were averaged after reverse scoring of the reverse questions, with higher mean values indicating stronger individual social support. The KMO value of the questionnaire was 0.972 and the Cronbach’s alpha coefficient of the questionnaire in this study was 0.924.

#### University students’ academic engagement scale questionnaire

The University Students’ Academic Engagement Scale Questionnaire was developed by Wang (2014) [69] in his doctoral dissertation. The scale contains five dimensions: active learning, teacher-student interaction, peer interaction, deep cognitive strategies and enthusiasm for learning. The first three dimensions belong to behavioral engagement, while the last two belong to cognitive and affective engagement, respectively. The questionnaire contains 22 items on a five-point scale. The scores of all items were averaged after reverse scoring the reverse questions, with higher mean values indicating higher individual academic engagement. The KMO value of the questionnaire was 0.972 and the Cronbach’s alpha coefficient of the questionnaire in this study was 0.932.

#### Adolescent student life satisfaction scale

The life satisfaction scale for adolescent students was developed by Zhang and He in 2004 [39]. Based on the Multidimensional Life Satisfaction Scale for Adolescents (MLSA) developed by Huebner (1994) [70], the scale was adapted to measure adolescent students’ learning and life. The life satisfaction scale for adolescent students is a self-report scale consisting of 6 dimensions of friendship, family, academics, freedom, school, and environment with 36 entries on a 5-point scale. The scores of all items were averaged after reverse scoring of the reverse questions, with higher mean values indicating higher individual life satisfaction. The KMO value of the questionnaire was 0.969 and the Cronbach’s alpha coefficient of the questionnaire in this study was 0.916.

#### University students’ academic motivation questionnaire

The academic motivation questionnaire for university students was developed by Tian and Pan in 2006 [39]. The questionnaire was based on Ozupal’s theoretical model of achievement motivation. The scale contains 4 dimensions of interest in knowledge, competence pursuit, reputation acquisition, and altruistic orientation, with 34 entries on a 5-point scale. The scores of all items were averaged after the reverse scoring of the reverse questions, with higher values indicating stronger individual academic motivation. The KMO value of the questionnaire was 0.976 and the Cronbach’s alpha coefficient of the questionnaire in this study was 0.93.

### Research procedures

Descriptive statistics and Pearson correlation analysis were performed in this study using SPSS 26.0. In order to ensure the accuracy of the results, the variance inflation factor (VIF) method was used in the study for the covariance test (if VIF > 10, it means that there is a serious covariance problem between the variables, and the corresponding variables need to be excluded). Meanwhile, the study used model 6 in the process plug-in prepared by Hayes (2017) [71] for chained mediation effect analysis and tested the significance of the mediation effect using the bias-corrected percentile Bootstrap method. It was considered statistically significant if the 99% confidence interval did not contain a value of zero [72]. In addition, before analyzing the data, a common method bias test was performed using the Harman single-factor test [73].

## Findings

### Common method bias test

When the self-report method was used to collect data, the issue of common method bias may arise. Therefore, the common method bias test was performed using the Harman single-factor test. The results showed that there were eight principal components with eigenvalues greater than 1. The first principal component explained 34% of the variance, which was below the critical criterion of 40%. Therefore, there was no serious common method bias in this study.

### Descriptive statistics and correlation analysis of the variables

Table 2 presented the mean and standard deviation of academic engagement, life satisfaction, social support, and academic motivation, as well as the Pearson product difference correlation coefficients between the variables. All the correlations between the variables all reached the significance level and could be further analyzed.

**Table 2:** Descriptive statistics and correlation analysis of the variables

	Mean standard	Deviation	Academic engagement	Life satisfaction	Social support	Academic motivation
Academic engagement	3.598	0.683	1			
Life satisfaction	3.504	0.562	0.838**	1		
Social support	3.632	0.712	0.738**	0.816**	1	
Academic motivation	3.586	0.703	0.695**	0.642**	0.655**	1

Note: ** *p* < 0.01

### Relationship between social support and academic engagement: a chain mediating model

#### Path coefficient analysis

**Fig. 1:**
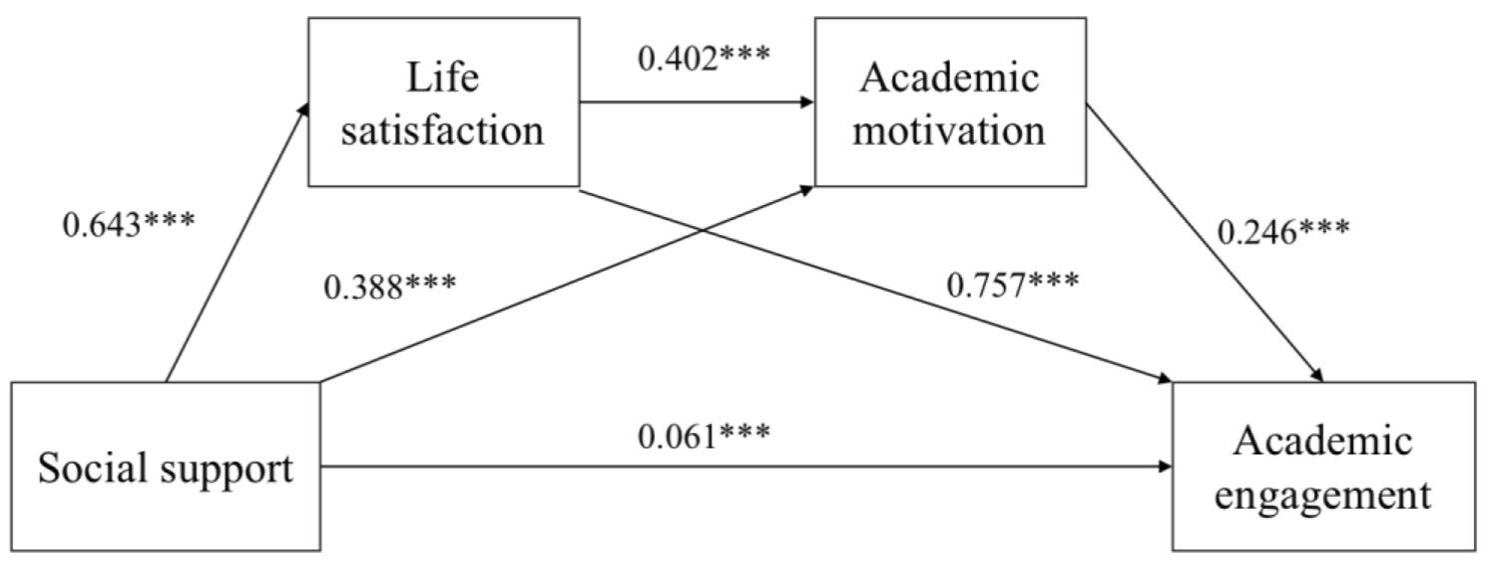
The chain mediation model (Note: *** *p* < 0.001)

The above analysis indicated a significant correlation between the variables and possible covariance. Therefore, before the effects were tested, the predictor variables in the equations were standardized and diagnosed for covariance. The results showed that the variance inflation factors for all the predictor variables (3.182, 3.277 and 1.865) were less than 5. Therefore, the data used in this study did not have serious covariance problems and were suitable for further tests of mediation effect. The process plug-in developed by Hayes was used to assess the 95% confidence interval (CI) for the mediating effect of life satisfaction and academic motivation in the effect of social support on students’ academic engagement (bootstrap sample size of 5000). The results of the chained mediation modeling were shown in Fig. 1; Table 3.

**Table 3:** The regression equation of chain mediation

Regression equation (N = 2106)		Fit indicator			Coefficient and significance	
Outcome variable	Predictor variable	${R^2}$	Adjustment${R^2}$	${F}$	$\beta$	${t}$
Life satisfaction	Constant	0.665	0.665	4184.757	1.168***	31.738
	Social support				0.643***	64.690
Academic motivation	Constant	0.464	0.463	910.022	0.768***	10.835
	Social support				0.388***	14.240
	Life satisfaction				0.402***	11.645
Academic engagement	Constant	0.545	0.545	2518.494	1.029***	19.729
	Social support				0.707***	50.185
Academic engagement	Constant	0.7632	0.5825	320.88	-0.159***	-3.266
	Social support				0.061***	3.217
	Life satisfaction				0.757***	31.716
	Academic motivation				0.246***	16.829

Note:***p < 0.001

The results showed that social support significantly and positively predicted academic engagement (β = 0.707, *p* < 0.001). With the addition of the mediating variables of life satisfaction and academic motivation, social support still significantly and positively predicted academic engagement, but with a significantly lower effect size (β = 0.061, *p* < 0.001). In addition, social support significantly and positively predicted life satisfaction (β = 0.643, *p* < 0.001) and academic motivation (β = 0.388, *p* < 0.001); life satisfaction significantly and positively predicted academic engagement (β = 0.757, *p* < 0.001); and academic motivation significantly and positively predicted academic engagement (β = 0.246, *p* < 0.001).

#### Mediation effect test

Further testing for mediating effects (see Table 4) found that the Bootstrap 95% CI intervals for the total indirect effects of life satisfaction and academic motivation in the effect of social support on academic engagement did not include zero. This indicated that life satisfaction and academic motivation were mediating variables in the effect of social support on academic engagement. Moreover, the total effect of social support on academic engagement was 0.707, with a direct effect of 0.061, accounting for 8.6% of the total effect, and a total indirect effect of 0.646, accounting for 91.4% of the total effect. This mediating effect was mainly composed of the following three paths:

1. Social Support -> Life Satisfaction -> Academic Engagement [95% CI = (0.432, 0.552), Boot SE = 0.025], with a mediating effect of 0.487, which accounted for 68.9% of the total effect, and Hypothesis 2 was supported;
2. Social Support -> Academic Motivation -> Academic Engagement [95% CI = (0.071, 0.130), Boot SE = 0.013], with a mediating effect of 0.095, which accounted for 13.4% of the total effect, Hypothesis 3 was supported;
3. Social Support -> Life Satisfaction -> Academic Motivation -> Academic Engagement [95% CI = (0.040, 0.101), Boot SE = 0.015], with a mediating effect of 0.064, which accounted for 9.1% of the total effect, and Hypothesis 4 was supported.

**Table 4:** Bootstrap analysis of the mediation effect test

	Effect	Boot SE	Boot LL CI	Boot UL CI	Relative mediation effect
Total	0.646				91.4%
Indirect effect 1	0.487	0.025	0.432	0.552	68.9%
Indirect effect 2	0.095	0.013	0.071	0.130	13.4%
Indirect effect 3	0.064	0.015	0.040	0.101	9.1%

Note:Ind1: Social Support -> Life Satisfaction -> Academic Engagement

Ind2: Social Support -> Academic Motivation -> Academic Engagement

Ind3: Social Support -> Life Satisfaction -> Academic Motivation -> Academic Engagement

## Discussion

### The effect of social support on academic engagement

The results of this study showed that social support positively predicts academic engagement, i.e., the group of university students with more social support will have higher degree of academic engagement, and conversely, the group of university students with less social support will have lower degree of academic engagement. This was consistent with the conclusions drawn from existing research. Social support can evoke positive psychological and behavioral responses by providing individuals with solutions to problems [74]. Students who receive a greater sense of social support are generally more likely to feel personally connected to the learning environment, to experience positive emotions in the classroom, and to actively use adaptive cognitive strategies for learning and participate better in learning tasks [37]. Students with more social support are better integrated into their support network and the university academic environment, thus increasing their academic achievement [75]. According to the social support theory, the provision of emotional, material, and informational support can enhance an individual’s ability and willingness to engage in specific behaviors [76]. When students receive more interactive support, they are more competent to deal with learning-related issues. Existing study shows the similar views [77]. These students believe that they can depend on their own ability to achieve their academic goals and see their studies as meaningful. For this reason, they can better persist in their studies and continue on a positive academic path [78]. Social support from parents and teachers helps develop students’ academic self-efficacy, which in turn promotes their academic engagement [79]. The social support from parents rises students’ awareness of the importance of education, promotes their desire for education and influences their academic attitudes and beliefs [80]. The social support from teachers can enhance students’ pursuit of mastery goals and interest in academic tasks, thus promoting their engagement in educational activities [81]. For example, teachers’ interaction with students during classroom activities, providing clear guidance and timely feedback to students can provide students with a sense of social support [32]. This helps deepen students’ sense of recognition with their schools and their understanding of learning value, at the same time regulating their stress and anxiety. For this reason, students’ academic engagement is positively influenced by teachers’ social support. Teachers with strong student-teacher relationships are more engaged with their students. They are able to employ strategies that engage students in deeper learning, which increases student engagement in academic activities [82]. In addition, other scholars have studied students’ online learning and reached similar conclusions. Students who receive more supportive responses and assistance for online learning invest more subjective efforts and self-regulated learning strategies in their learning activities, which increase their level of academic engagement [36].

### Mediating effect of life satisfaction

The results of this study showed that life satisfaction plays a partial mediating role between social support and academic engagement. That is, the group of university students with more social support has higher level of life satisfaction, and thus the degree of their academic engagement will be higher. This is similar to the conclusions reached by existing studies. Lin et al. (2020) note that people’s perception of social support protects them from negative outcomes and makes them less vulnerable to mental illness [83]. Additionally, individuals with more social support can more effectively cope with challenges to physical health and are more likely to overcome obstacles. For example, students who receive more support from teachers more experience joy, interest, and hope in learning and thus less anxiety, depression, or despair [84, 85]. In the face of stress, frustration, and challenges, such students are able to actively utilize the available social resources as an alternative method to overcome emotional imbalances [86]. Social support can help individuals cope with learning demands and provides them with stronger beliefs and motivation to adapt to their learning [43]. A study of Chilean university students has found that plummeting levels of social support exacerbated the frustration of adolescent students, thereby reducing their life satisfaction. This leads to students to be prone to pessimism about learning and not to believe their ability to meet the expectations of their teachers and parents on learning, thus decreasing academic engagement [87]. More social support helps individuals better adapt to the new and changing social environments, reduce stress-induced tension, and thus enable students to experience higher level of life satisfaction [40, 42]. Social support enhances emotional resilience in students. Close relationships with friends help adolescents better control their emotions, provide them with emotional support, and encourage self-expression and self-discovery in a stable environment, thus increasing individuals’ life satisfaction [43]. At the same time, a sense of social support can help bring about positive self-focused thinking. Social support from significant sources leads individuals to generate positive self-evaluations and develop other related personal traits, such as positive self-focus and self-concept, and subsequently leads them to have positive life experiences (e.g., life satisfaction) [88]. The higher the level of students’ life satisfaction is, the more engaged they are in learning. Social support from teachers and peers, as well as parents, can help students’ meet the basic needs for competence (i.e., a sense of effectiveness and mastery of learning), autonomy (i.e., freedom or ownership in learning), and relevance (i.e., a sense of belonging and a sense of connection to teachers and peers). Students tend to be emotionally, behaviorally, and cognitively engaged in a learning task when teachers support their independent learning [89]. When these basic needs are met, students’ life satisfaction improves and thus their engagement in academic activities is promoted [90]. A study has confirmed that more social support for adolescents would correspondingly increase their life satisfaction. They will have a more positive attitude towards their lives and be more interested in their academic and school activities, thus focusing more on their academic tasks [46]. A study of students with dyslexia has found that this type of students were exposed to fewer resources of professional tutors, leaving them with little knowledge about the options that they can have to receive substantial help. When students receive lower social support, their life satisfaction is accordingly affected. They often experience secondary emotional and motivational barriers, which in turn reduces their academic engagement [91]. In conclusion, the social support that university students receive can help them cope with the challenges from their studies, meet their basic needs and reduce negative psychological impact, thus enhancing their life satisfaction. The higher their life satisfaction is, the more they are inclined to engage in their studies.

### Mediating effects of academic motivation

The results of this study showed that academic motivation plays a partial mediating role between social support and academic engagement. That is, the more the social support is, the stronger the academic motivation is, and thus the higher degree of the academic engagement becomes. This finding has been confirmed by existing studies. With today’s students facing increasing levels of anxiety, parents, teachers, and other educational professionals can help students equip with coping strategies to anxiety, thereby promoting their mental health and ultimately their academic motivation [92]. Ryan & Deci (2020), based on self-determination theory, argue that authentic, warm, and supportive environments provided by teachers and peers help students meet basic psychological needs (e.g., relatedness, etc.), which increases internal motivation to learn [89]. Students tend to have stronger academic motivation when they perceive that their teachers and peers clearly communicate expectations and values that are consistent with their own interests and provide resources and assistance, including emotional support, that are needed to fulfill those expectations and values [93]. Previous studies hold the similar view [94, 95]. A study of English language learning among university students has found that teachers who provide social support to their students tend to create learner-centered environments and attempt to understand the emotional state of their learners, which increases students’ academic motivation. This can help students deal with challenging problems and reduce their mental stress [96]. Students who receive a higher sense of social support from their peers tend to have clearer plans and higher expectations for their own academics because of the positive motivational feedback they feel, thus promoting their own learning progress [97]. Academic engagement begins when students actively acquire knowledge based on internal motivation to learn and activate the cognitive processes required for successful problem solving [98]. Students with high levels of internal motivation are more aware of themselves and standardize their study plans, which increases the amount of effort they put into their studies [99]. Students with high degree of academic motivation may take advantage of learning opportunities in pursuit of their learning goals in order to perform better than others, rather than simply to acquire knowledge, thus increasing their degree of academic engagement [100]. This kind of students tends to become more resilient in their motivation. They are able to adjust their academic strategies in accordance with their own learning situations, take the initiative and focus on learning, and thus have a high degree of academic engagement [101]. They are able to adopt more adaptive coping strategies (particularly strategizing, help-seeking, and self-encouragement) when faced with difficult challenges, which in turn increases the degree of academic engagement [102, 103]. Students’ motivated persistence promotes the adoption of academic strategies and provides feedback on their learning outcomes, which in turn is more conducive to subsequent academic engagement [104]. Individuals receive various types of information through social support, including the fact that they believe to be appreciated and liked by others and the fact that they believe to be valued and a part of a social network, which fully mobilizes the individual’s academic motivation and enables them to engage in learning tasks spontaneously [105]. When students are supported by their teachers, they are more likely to attend class and develop close relationships with their teachers, which increases their academic motivation. This in turn helps students to increase their self value, social self-esteem and the feeling that they can take control of their lives, which in turn promotes their level of academic engagement [106]. The more social support university students receive, the more positive feedback and incentives they receive, the stronger their level of academic motivation is. And with their increased motivation level, they are more inclined to engage in academic learning.

### Chain mediation effect of life satisfaction and academic motivation

This study found that life satisfaction and academic motivation have a close relationship, and the two constitute an intermediate link in the influence path of social support -> life satisfaction -> academic motivation -> academic engagement, which has a chain mediation effect in the influence of social support on academic engagement. That is, university students with more social support have higher level of life satisfaction, which leads to stronger academic motivation and consequently higher degree of academic engagement. Social support is a buffer for university students when they are faced withf stress and adversity. Social support allows individuals to feel cared, valued, and even to have a contact in case of an emergency [107]. It helps individuals to reassess stressors as less threatening, which in turn facilitates the development of problem-solving strategies. Therefore, social support has a positive predictive effect on life satisfaction [108]. Students with high life satisfaction have the confidence to make necessary efforts to succeed, persevere in achieving their academic goals and are able to overcome the setbacks they face [48, 109]. Students who receive social support are able to increase their life satisfaction by solving problems in a positive manner, finding appropriate ways to improve the current situation or to prevent the stressful events from recurring in the future and by encouraging themselves to regulate their emotions constructively. These coping styles allow students to return to academic activities with new energy and strategies for approaching tasks, improving the learning environment and thus greatly increasing the degree of student academic engagement [110]. Meanwhile, pre-service teachers are more likely to learn effectively in classrooms where teacher educators provide clear instructions, instrumental support and constructive feedback, support learning autonomy, and promote collaborative learning. When pre-service teachers’ needs for competence or effective learning are met, they are internally motivated to learn and adopt better knowledge integration strategies, and thus are more likely to be cognitively, emotionally, and behaviorally engaged in learning [58]. Opdenakker (2021) suggest that when students feel socially supported, they are able to gain a sense of identity in their interactions with the social environment, have more opportunities to express and expand their abilities, and thus aspire to further academic development, which fully mobilizes academic motivation that in turn increases their attention and concentration on classroom activities [111]. In addition, a number of studies have confirmed that life satisfaction is positively correlated with academic motivation. high level of life satisfaction can correspondingly increase students’ basic academic expectations and balance negative emotions such as academic irritation through, for example, rational self-regulation [112]. This kind of students are able to promote the ability to understand and regulate their own and others’ emotions and can better overcome frustrations encountered in their academic life, thus showing stronger academic motivation [113]. This is in line with existing studies. Individuals with higher life satisfaction are able to engage in more stable self-regulation and can better internalize external demands, which in turn helps individuals to acquire greater learning autonomy and better learning outcomes with stronger academic motivation [67]. Students with higher life satisfaction possess skills that make them feel empowered to achieve their goals, take control of their lives and take responsibility for their outcomes, and thus have more motivation to learn [114]. These students tend to face life with full positive emotions. This facilitates to promote meta-cognitive thinking and the use of creative learning strategies to achieve their goals [115]. They are able to enhance their academic motivation in environments characterized by a sense of safety and closeness, allowing them to persist and engage in selected tasks for longer periods of time, which in turn promotes positive learning outcomes [116]. Therefore, the mediating roles of life satisfaction and academic motivation should be given consideration when exploring the mechanisms by which social support influences academic engagement is explored.

## Conclusions

This study examined the mechanism of how social support’s influences on university students’ academic engagement, and the chain mediating role of life satisfaction and academic motivation in the mechanism. This study found (1) social support significantly and positively predicts academic engagement; (2) social support influences academic engagement through the mediating effect of life satisfaction; (3) social support influences academic engagement through the mediating effect of academic motivation; (4) life satisfaction and academic motivation play a chain mediating role in the effect of social support on academic engagement.

These findings can help understand the inner mechanism of the relationship between social support and academic engagement, which in turn provides insights for universities and the departments concerned to improve the level of university students’ academic engagement. Our society should better understand the social support that students receive from teachers, peers, and guardians and build supportive learning environments as equal as possible for them [117]. Universities should try their best to create a comfortable accommodation environment, a safe food environment and a free and harmonious interpersonal atmosphere for students, and offer colorful extracurricular activities, so as to improve the life satisfaction of university students. At the same time, universities can help students understand the significance of learning and clarify their career development path through relevant courses and lectures, thus stimulating their academic motivation, reducing learning burnout, and enabling them to be more actively engaged in learning. On a daily basis, teachers should make efforts to improve the quantity and quality of social support provided and to facilitate interactions between students [118]. Students get more appreciation and praise from teachers and peers, which enhances their self-efficacy, stimulates their interest in learning, and makes them better complete learning tasks. Parents should also give more positive guidance to university students, provide them with appropriate financial and mental support for their studies, and help them overcome all kinds of obstacles encountered in the process of learning. Through the concerted efforts of all parties, we can jointly improve the level of university students’ academic engagement.

## Contributions, limitations and prospects

### Contributions

There have been many researches on university students’ social support, which is of great significance to their healthy development. Social support is a buffer against stress [119], which can improve an individual’s psychological state [120], and increase an individual’s perception of his or her own value [121]. Social support also provides university students with a sense of security and competence [122]. Among them, there are also many studies concerned with the influence of social support on university students’ academic engagement [36, 37]. However, there are relatively fewer separate investigations into the mediating roles of life satisfaction [110] and academic motivation [111] in this process, and extremely few ones that have simultaneously explored the chain mediating role of the two in the influence of social support on academic engagement. This study systematically and comprehensively explores the influence mechanism of social support on university students’ academic engagement. This study to a certain extent enriches the theoretical research on the influence mechanism of university students’ social support on university students’ academic engagement, which is instructive for the subsequent research. At the same time, the conclusions drawn from this study also provide references for universities and the departments concerned to improve the degree of university students’ academic engagement at the practical level, which in turn can promote the quality of talent cultivation in undergraduate universities.

### Limitations and prospects

This study investigated the influence mechanism the social support on university students’ academic engagement by surveying 2,106 university students from different undergraduate universities across Chins through the principle of convenience sampling. The study has certain contributions at both the theoretical and practical levels. Of course, this study still has some limitations. First, the sample was limited by the cross-sectional data sources and remains deficient in the confirmatory nature of the causal inferences of the variables. Due to the constraints on the authors’ time and energy, only one collection of data was conducted in this study. In subsequent research, longitudinal studies can be conducted in context, with multiple collections of data tracking the development of the mechanism by which social support influences universities students’ academic engagement over time. Second, there may be selection bias and potential threats in the case of convenience sampling. Future research could adopt the method of multiple data collections. Finally, due to time constraint, the questionnaire was administered without intervention. The follow-up study could add appropriate interventions into the questionnaire process.

## Data Availability

The raw data supporting the conclusions of this article will be available from Chunmei Chen (chunmei88@jmu.edu.cn) on resonable requests.
